# A novel system for intercostal nerve cryoablation during robotic hybrid ablation

**DOI:** 10.1016/j.xjtc.2025.05.008

**Published:** 2025-05-30

**Authors:** Zain Khalpey, Ujjawal Kumar, Tyler Phillips, Rahul Doshi, Yoaav Krauthammer

**Affiliations:** a Department of Cardiac Surgery, HonorHealth, Scottsdale, Ariz; b Khalpey AI Lab, Applied & Translational AI Research Institute, Scottsdale, Ariz; c School of Clinical Medicine, University of Cambridge, Cambridge, United Kingdom; d Department of Electrophysiology, HonorHealth, Scottsdale, Ariz

**Figure:**
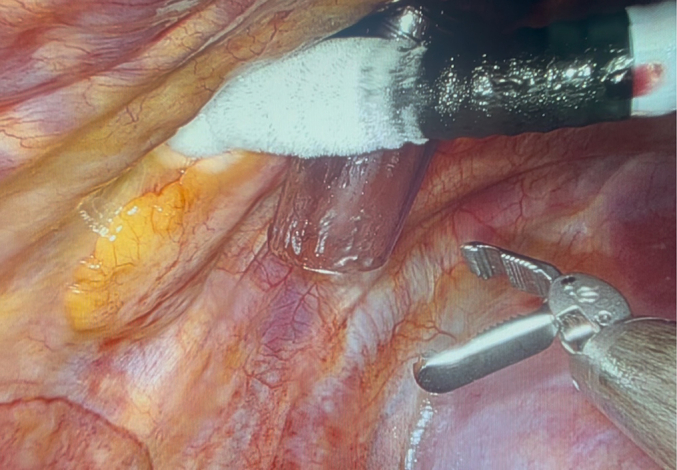
Intercostal nerves 4 to 7 were cryoablated close to the posterior axillary line.

**Central Message**

Intercostal nerve cryoablation is an effective adjunct for postoperative analgesia. We report the first application of a novel system in robotic surgery, resulting in negligible postoperative pain.

## Introduction

Intrathoracic surgery has a high potential for postoperative pain due to the manipulation of skeletal, muscular, and neuropathic systems, impairing pulmonary and functional rehabilitation. Enhanced recovery protocols promote opioid-sparing strategies with adjuncts such as intercostal nerve cryoablation.^1^ This reduces nerve temperature, degenerating the axon while preserving the nerve sheath and connective tissue to disrupt pain signals while preserving nerve structure.^2^ It has previously shown efficacy in various cardiothoracic procedures.^3^ We present the first use of a novel cryoablation system in robotic surgery.

## Case presentation

A 78-year-old man with long-standing, persistent atrial fibrillation (AFib) despite catheter ablation was evaluated for robotic hybrid ablation. Preoperative assessments showed no myocardial ischemia, normal left ventricular ejection fraction, moderate left ventricular hypertrophy, mild mitral and tricuspid regurgitation, and pulmonary hypertension (right ventricular systolic pressure of 58 mm Hg).

**Figure 1:**
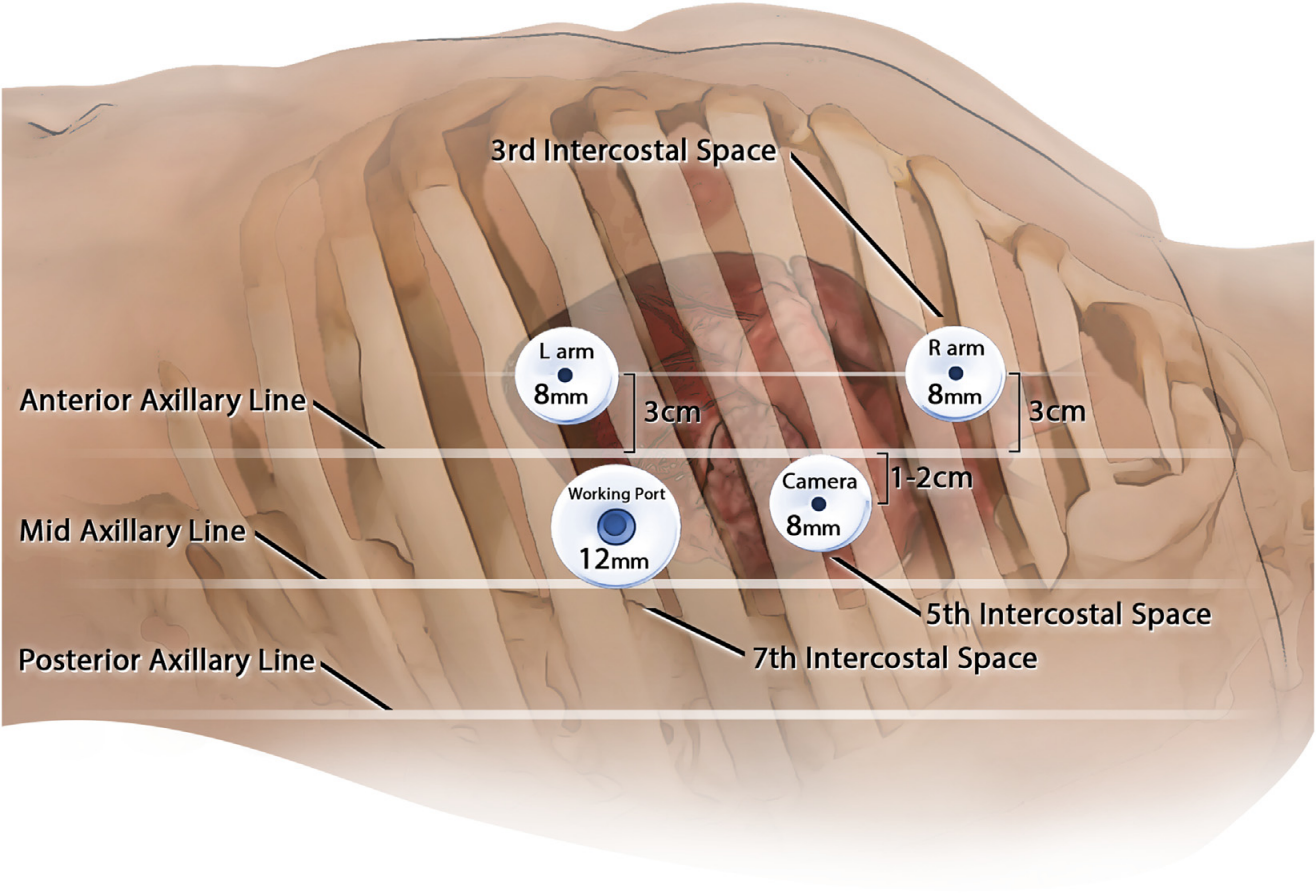
Operative setup.

**Figure 2:**
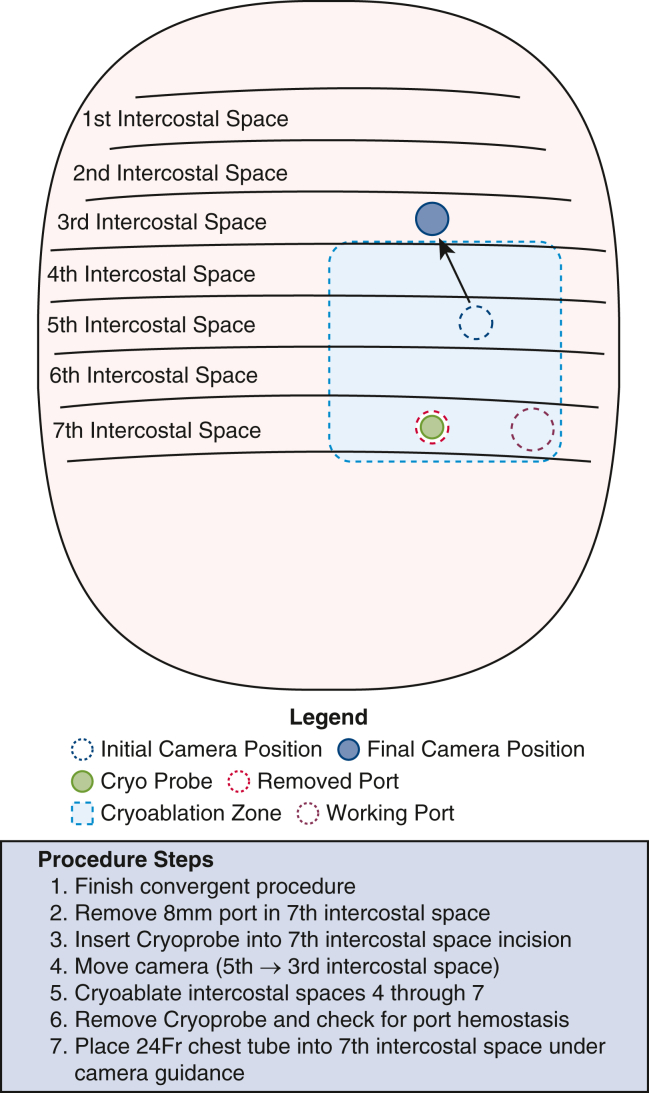
Procedure for intercostal nerve cryoablation.

A full description of our perioperative clinical protocol is given in Appendix E1. Incisions were made, ports were introduced, and the da Vinci Xi robot (Intuitive) was docked (Figure 1). The patient, initially in mixed AFib/atrial flutter, underwent epicardial mapping (EnSite Precision, Abbott). A total of 23 lesions were made, and the left atrial appendage was excluded.^4^ The seventh interspace port was removed (Figure 2), the cryoprobe (CryoSMax-L, AtriCure) was bent to 80°, and the fourth to seventh intercostal spaces were cryoablated close to the posterior axillary line for 60s at −70 °C. After cryoablation, the cryoprobe was removed, and a 24F chest tube was placed. Electrocardiography showed AFib, although direct current cardioversion restored an organized normal sinus rhythm (NSR). Remaining ports were removed, intercostal nerve blocks were performed, local anesthesia was infiltrated around the incisions, and the patient was extubated in the operating room without complication.

Postoperatively, the patient had minimal pain (tracked using the document in the Online Data Supplement) and was discharged on postoperative day (POD) 2. He reported incisional numbness with moderate shoulder and chest tube site pain on POD 1, but achieved a satisfactory 1750 mL on incentive spirometry. His total opioid use was minimal, with 2 morphine milligram equivalents required on POD 1 (0.2 mg hydromorphone every 12 hours) and 1 morphine milligram equivalent required on POD 2 (0.2 mg hydromorphone). Dynamic pain scores (upon deep breathing, coughing, ambulation, and full-power upper-limb movements) were negligible at discharge.

At 30-day follow-up, when presenting for endocardial ablation, he was satisfied with pain management, requiring no opioids after hospital discharge. He had no chest wall pain with returning sensation and residual numbness anteriorly from the medial aspect of the nipple to the sternum. He was in NSR, with left atrial appendage closure confirmed using transesophageal echocardiography. Endocardial ablation was performed, and he was discharged that day in NSR.

## Comment

Intercostal nerve cryoablation is an effective pain management adjunct, facilitating opioid reduction and enhanced recovery through optimization of postoperative analgesia.^1^^,^^2^ This case highlights the feasibility and effectiveness of the CryoSMax-L system for intercostal nerve cryoablation in robotic surgery.

By lowering nerve temperature to −70 °C, cryoablation induces reversible axonotmesis, disrupting nerve conduction^2^ and providing lasting analgesia. In this case, it helped address pain from multiple incisions and port placements with minimal opioids. Low dynamic pain scores and satisfactory incentive spirometry values suggest its potential to mitigate postoperative pulmonary complications in cardiothoracic patients. Patient satisfaction and absence of pain at 30 days further support the potential value of cryoanalgesia as a tool within the perioperative analgesic armamentarium for robotic cardiothoracic surgery.

CryoSMax-L uses nitrous oxide (rather than carbon dioxide) with a 60% greater contact area, improving axonal disruption consistency. Previous probes often achieved incomplete axonotmesis, contributing to neuropathic pain in some studies, such as a recent randomized controlled trial.^5^ Although effective, cryoanalgesia can result in intercostal numbness, which may be bothersome to some patients.

Most research on cryoablation focuses on thoracotomy. Robotic surgery, with its lack of haptic feedback and greater port torque, presents a distinct pain profile with a potential role for cryoanalgesia. Although we report our technical approach and satisfactory outcome, comparing it with other approaches is beyond the scope of this work. Further studies are warranted and ongoing to assess sensory recovery, patient-reported outcomes, and the role of cryoanalgesia in multimodal pain management strategies for robotic-assisted cardiothoracic surgery. Our group is concurrently collecting longitudinal neuropathy and functional recovery data to evaluate the duration and impact of cryoanalgesia-related sensory changes.

## Conflict of Interest Statement

The authors reported no conflicts of interest.

The *Journal* policy requires editors and reviewers to disclose conflicts of interest and to decline handling or reviewing manuscripts for which they may have a conflict of interest. The editors and reviewers of this article have no conflicts of interest.

## Figures and Tables

**Table E1:** Preoperative medications

Medication	Dose	Route	Timing	Indication
Methylprednisolone (Solu-Medrol)	125 mg	IV	∼30-60 min preincision	Reduces systemic inflammatory response to ablation
Famotidine	20 mg	IV	Preoperative area	Esophageal mucosal protection; reduces acid exposure in case of injury; also provides protection against NSAID or steroid-induced gastritis
Gabapentin (as per ERAS protocol)	300-600 mg	PO	1-2 h preoperatively	Multimodal analgesia to reduce opioid requirements
Antibiotic prophylaxis	Cefazolin 2 g (per local protocol)	IV	≤60 min before incision	Surgical site infection prevention

*IV*, Intravenous; *PO*, per os, oral; *NSAID*, non-steroidal anti-inflammatory drug.

**Table E2:** Intraoperative medications

Medication/intervention	Dose/details	Route	Timing	Indication
Intercostal block and local anesthesia	3-5 mL of 0.5% bupivacaine (Marcaine) without epinephrine for each of the 4 nerve blocks, and a total of up to 5 mL for all 4 port sites	Local infiltration	Before closure	Analgesia for intercostal pain from robotic port access
Cryoablation	−70 °C for 1 min at each site	Direct application	After ablation + LAAL, before chest tube	Adjunct for intercostal pain from robotic port access
Esophageal probe	Circa S-CATH M esophageal temperature probe	Transesophageal	Throughout	Prevent thermal injury to the esophagus and atrioesophageal fistulization

*LAAL*, Left atrial appendage ligation.

**Table E3:** Postoperative medications

Medication	Dose	Route	Timing	Indication
Hydrocodone/acetaminophen	7.5 mg/325 mg	PO	As required, maximum every 4 h	Pain control postprocedure
Ketorolac (Toradol)	15-30 mg	IV	Single dose in PACU or ICU	Anti-inflammatory effect; postoperative pain control
Colchicine	0.5 mg	PO	Begin within 24 h postoperatively, continue for 3 to 5 d, every 12 h	Postpericardiotomy pericarditis prophylaxis
Medrol pack (methylprednisolone taper)	See below	PO	Start on postoperative day 1	Anti-inflammatory taper for ongoing control of pericardial/systemic inflammation

Medrol Taper Schedule. • Day 1: 6 tablets (24 mg) divided doses • Day 2: 5 tablets (20 mg) divided doses • Day 3: 4 tablets (16 mg) divided doses • Day 4: 3 tablets (12 mg) divided doses • Day 5: 2 tablets (8 mg) divided doses • Day 6: 1 tablet (4 mg) in the morning

*PO*, Per os, oral; *IV*, intravenous; *PACU*, post anesthesia care unit; *ICU*, intensive care unit.

**Table E4:** Discharge medications

Medication	Dose	Route	Duration	Indication
Hydrocodone/acetaminophen	7.5 mg/325 mg, maximum every 4 h	PO	As required	Pain control postprocedure
Colchicine	0.5 mg, every 12 h	PO	3-5 d	Postpericardiotomy pericarditis prophylaxis
Medrol dose pack	As above	PO	6-d taper	Anti-inflammatory coverage
Famotidine	20 mg, every 12 h	PO	Until steroids complete	Gastric protection if steroids/NSAIDs continued
Anticoagulation	Resume preoperative DOAC/warfarin	PO	As soon as bleeding risk allows	Stroke prevention postablation
Antiarrhythmic drugs	Resume/initiate per EP protocol	PO/IV	As directed	Rhythm control strategy postablation

*PO*, Per os (oral); *NSAID*, nonsteroidal anti-inflammatory drug; *DOAC*, direct oral anticoagulant; *EP*, electrophysiology; *IV*, intravenous.
