# Supplementary material for: A novel system for intercostal nerve cryoablation during robotic hybrid ablation
Source: JTCVS Tech. 2025 May 30;32:91–5. doi: 10.1016/j.xjtc.2025.05.008 (PMC12347727; doi:10.1016/j.xjtc.2025.05.008)
Supplement: Online Data Supplement [file mmc1.pdf]

PATIENT STICKER

Cryo Nerve Block
Patient Pain Tracking

General

Hospital: ______________ Surgeon: ______________ Date of Surgery: ______________

Surgery Type:

Cardiac: AVR MVR TVR CABG ASD VSD PDA PFO Arch Repair

Ascending Aortic Descending Aortic Maze Decoarctation Other: ______________

Thoracic: Pneumonectomy Lobectomy Wedge Resection Segmentectomy Lung Volume Reduction

Lung Transplant Pectus Excavatum Repair Pectus Carinatum Repair Rib Fracture Repair

Esophagectomy Resection of Chest Wall Tumor Other: ______________

Procedural Approach: Thoracotomy VATS Robotic Sternotomy

Procedural Site: Right Left Bi-Lateral

Were medication induced nerve block performed on the patient? Yes No

If yes, when were they performed: Prior to incision After procedure

If yes, what was given: Marcaine Ropivacaine Exparel Other: ______________

cryoNB surgical approach: Extrathoracic Intrathoracic

When was cryoablation performed: Prior to index procedure Middle of index procedure End of index procedure

Incision levels (Trocar/Thoracotomy):

Left:	1	2	3	4	5	6	7	8	9	10	11
Right:	1	2	3	4	5	6	7	8	9	10	11

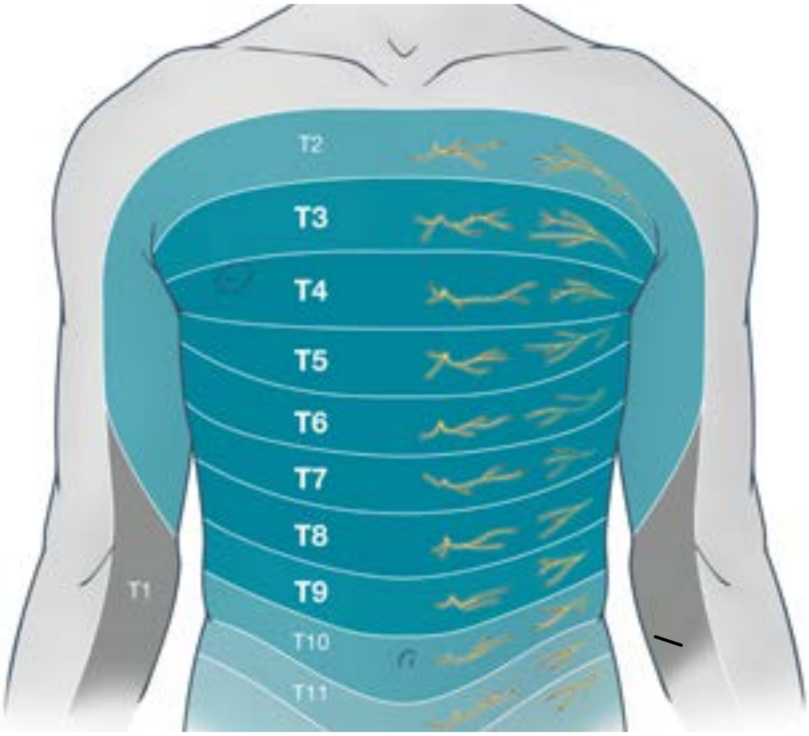

AtriCure is providing this patient pain tracking form as an example template to be completed by internal hospital staff only.
Prior to using this form for other purposes, please consult your Internal Review Board.

POD1 ASSESSMENT

Date:

Pain Score (AM): ________ 1 through 10 *(10 is worst pain)*

Pain Score (PM): ________ 1 through 10 *(10 is worst pain)*

Pain at Level of Incision(s): Y N

Sharp | Dull | Numb | Burning | None

Pain Outside the Incision: Y N

If Y, Describe: ________________

Inc Spirometry: ________ ml

Frequency Goal: Met | Not Met

Volume Goal: Met | Not Met

Deep Breathing: Y N

Cough: Y N

Taking Pain Meds: Y N

If Y:

Opiate Medication: ________________

Route of Administration: ________________

Opiate Dose w/ Units: ________________

Frequency: ________________

— or —

Total Dose: ________________

Ambulation

Frequency Goal: Met | Not Met

Distance Goal: Met | Not Met

POD2 ASSESSMENT

Date:

Pain Score (AM): ________ 1 through 10 *(10 is worst pain)*

Pain Score (PM): ________ 1 through 10 *(10 is worst pain)*

Pain at Level of Incision(s): Y N

Sharp | Dull | Numb | Burning | None

Pain Outside the Incision: Y N

If Y, Describe: ________________

Inc Spirometry: ________ ml

Frequency Goal: Met | Not Met

Volume Goal: Met | Not Met

Deep Breathing: Y N

Cough: Y N

Taking Pain Meds: Y N

If Y:

Opiate Medication: ________________

Route of Administration: ________________

Opiate Dose w/ Units: ________________

Frequency: ________________

— or —

Total Dose: ________________

Ambulation

Frequency Goal: Met | Not Met

Distance Goal: Met | Not Met

POD3 ASSESSMENT

Date:

Pain Score (AM): ________ 1 through 10 *(10 is worst pain)*

Pain Score (PM): ________ 1 through 10 *(10 is worst pain)*

Pain at Level of Incision(s): Y N

Sharp | Dull | Numb | Burning | None

Pain Outside the Incision: Y N

If Y, Describe: ________________

Inc Spirometry: ________ ml

Frequency Goal: Met | Not Met

Volume Goal: Met | Not Met

Deep Breathing: Y N

Cough: Y N

Taking Pain Meds: Y N

If Y:

Opiate Medication: ________________

Route of Administration: ________________

Opiate Dose w/ Units: ________________

Frequency: ________________

— or —

Total Dose: ________________

Ambulation

Frequency Goal: Met | Not Met

Distance Goal: Met | Not Met

SURGICAL FOLLOW-UP ASSESSMENT

Date:

Pain Score (AM): ________ 1 through 10 *(10 is worst pain)*

Pain Score (PM): ________ 1 through 10 *(10 is worst pain)*

Pain at Level of Incision(s): Y N

Sharp | Dull | Numb | Burning | None

Pain Outside the Incision: Y N

If Y, Describe: ________________

Inc Spirometry: ________ ml

Frequency Goal: Met | Not Met

Volume Goal: Met | Not Met

Deep Breathing: Y N

Cough: Y N

Taking Pain Meds: Y N

If Y:

Opiate Medication: ________________

Route of Administration: ________________

Opiate Dose w/ Units: ________________

Frequency: ________________

— or —

Total Dose: ________________

Ambulation

Frequency Goal: Met | Not Met

Distance Goal: Met | Not Met
